# Pre-transplant immune profile defined by principal component analysis predicts acute rejection after kidney transplantation

**DOI:** 10.3389/fimmu.2023.1192440

**Published:** 2023-07-11

**Authors:** Emilie Gaiffe, Mathilde Colladant, Maxime Desmaret, Jamal Bamoulid, Franck Leroux, Caroline Laheurte, Sophie Brouard, Magali Giral, Philippe Saas, Cécile Courivaud, Nicolas Degauque, Didier Ducloux

**Affiliations:** ^1^ Besançon University Hospital, INSERM CIC-1431, Besançon, France; ^2^ Univ. Franche-Comté, INSERM, Etablissement Français du Sang Bourgogne Franche-Comté, Unité Mixte de Recherche (UMR) 1098, RIGHT Interactions Hôte-Greffon-Tumeur/Ingénierie Cellulaire et Génique, Besançon, France; ^3^ Besançon University Hospital, Department of Nephrology, Besançon, France; ^4^ Centre Hospitalier Universitaire (CHU) Nantes, Nantes Université, INSERM, Center for Research in Transplantation and Translational Immunology, Unité Mixte de Recherche (UMR) 1064, Institut de Transplantation Université de Nantes (ITUN), Nantes, France

**Keywords:** immune profile, biomarker, acute rejection, kidney transplantation, hierarchical clustering analysis

## Abstract

**Background:**

Acute rejection persists as a frequent complication after kidney transplantation. Defining an at-risk immune profile would allow better preventive approaches.

**Methods:**

We performed unsupervised hierarchical clustering analysis on pre-transplant immunological phenotype in 1113 renal transplant recipients from the ORLY-EST cohort.

**Results:**

We identified three immune profiles correlated with clinical phenotypes. A memory immune cluster was defined by memory CD4^+^T cell expansion and decreased naïve CD4^+^T cell. An activated immune cluster was characterized by an increase in CD8^+^T cells and a decreased CD4/CD8 ratio. A naïve immune cluster was mainly defined by increased naïve CD4^+^T cells. Patients from the memory immune profile tend to be older and to have diabetes whereas those from the activated immune profile were younger and more likely to have pre-transplant exposure to CMV. Patients from the activated immune profile were more prone to experience acute rejection than those from other clusters [(HR=1.69, 95%IC[1.05-2.70], p=0.030) and (HR=1.85; 95%IC[1.16-3.00], p=0.011). In the activated immune profile, those without previous exposure to CMV (24%) were at very high risk of acute rejection (27 vs 16%, HR=1.85; 95%IC[1.04-3.33], p=0.039).

**Conclusion:**

Immune profile determination based on principal component analysis defines clinically different sub-groups and discriminate a population at high-risk of acute rejection.

## Introduction

With progress in immunosuppression, acute rejection became less frequent during the last decades. However, it still concerns 15 to 20% of kidney transplant recipients and affects long-term graft survival (1, 2). Pre-transplant risk factors explaining why only some patients developed acute rejection while they are all exposed to similar immunosuppression are imperfectly defined. Clinical factors (age, race), Human Leucocyte Antigen (HLA) typing and alloantibody screening are practically the only determinants that can be used for risk stratification.

In this context, it is tempting to search for immune biomarkers that could be predictive of acute rejection. Several candidates have been evaluated. Patients with positive donor-reactive or panel T cell reactive IFNγ ELISPOT assay are more likely to experience acute rejection (3, 4). Pre-transplant soluble CD30 (Cluster of Differentiation) has been suggested to be predictive of acute rejection (5). Our group reported that pre-transplant Recent Thymic Emigrant (RTE) were strongly associated with the occurrence of acute rejection in antithymocyte globulin (ATG)-treated kidney transplant recipients (6). Finally, the incidence of acute rejection has been reported to depend on specific genetic polymorphisms (7–9). Nevertheless, it should be emphasized that most of these studies have not or could not be replicated.

All these studies suffered from a major bias. Every immune parameter, cell or molecule, interacts with virtually all the immune system and should be interpreted in a general context, taking into account for positive and negative interactions. Thus, a more global approach is needed to consider the complexity of the system. Nevertheless, the number of information to collect is very important and often redundant. Thus, the data have to be summarized and reduced for better analysis.

Principal component analysis (PCA) is a method of comprehensive multivariate statistics and data analysis that allows to reduce the dimensionality of a dataset, enhancing interpretation while preserving information diversity (10). In the clinical setting of kidney transplantation, PCA should permit to merge patients with a similar biological profile. Thus, detection of specific immune profiles would be critical for detection of at-risk patients for acute rejection and subsequent targeted prevention.

In this study, we used individual determination of a panel of immune cells obtained from flow cytometry (including both frequency and total amount). Using hierarchical clustering, we separated different groups of patients based on immune profile. We first correlated these biological profiles with clinical profiles and second, determined whether biological profile may help to discriminate patients at risk for acute rejection.

## Materials and methods

### Patients

Research has been conducted in the 1113 kidney transplant recipients from the *Influence de l’Orientation de la Réponse LYmphocytaire* (ORLY-Est, NCT02843867) study. ORLY-Est is a prospective cohort study of incident renal transplant recipients in 7 French transplant centers (Besançon, Clermont-Ferrand, Dijon, Le Kremlin-Bicêtre, Nancy, Reims and Strasbourg) (6). The main objective of this study was to describe interactions between immune status and atherosclerosis after transplantation. For each patient, blood samples were collected at the time of transplantation and 1 year later. Clinical data were prospectively collected at the time of transplantation, 1 year, 3 years, 5 years and 10 years later. Sample collection was performed after regulatory approval by the French Ministry of Health (agreement number DC-2008-713, June 11, 2009). The ethics committee of the Franche-Comté study approved the study (2008). Patients enrolled in the ORLY-Est study gave their written informed consent.

**Figure 1:**
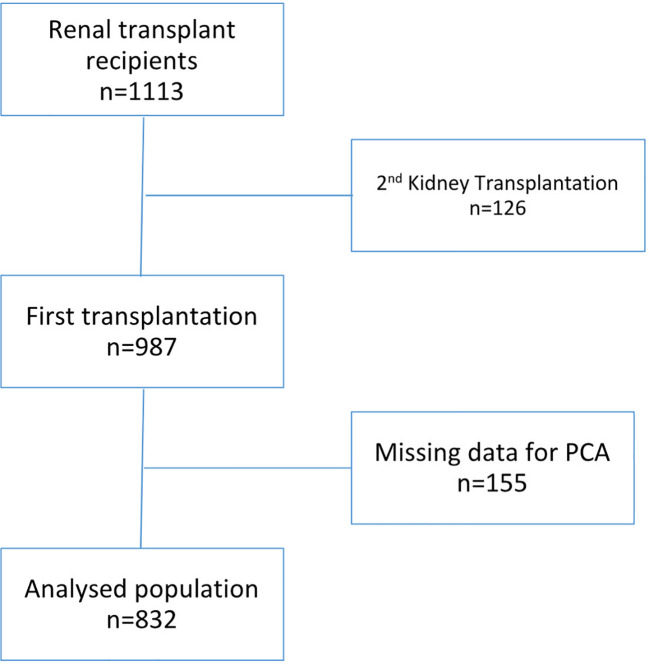
Patient flow chart.

To avoid the effects of previous immunosuppression on immune profile, we excluded patients having received a previous transplantation (n=126, 11.3%). Among the remaining 987 recipients of a first transplant, 205 patients (21%) had received T-cell depleting ATG therapy and 528 (63%) had received nondepleting aCD25 mAb therapy. One hundred and fifty five (15.7%) had missing data making inclusion in PCA impossible. Finally, eight hundred and thirty two were analyzed (**Figure 1**). Calcineurin inhibitors and mycophenolate mofetil were widely used as an immunosuppressive regimen. All the transplant procedures were performed with a negative cross match. Cytomegalovirus (CMV) prophylaxis was given according to each center’s practice. All patients received Pneumocystis antimicrobial prophylaxis with trimethoprim sulfamethoxazole for at least 6 months.

### Confounding factors

Age, gender, body mass index, diabetes, dyslipidemia, hypertension, past history of cardiovascular events, previous neoplastic history, and chronic lung disease were analyzed as covariates. Dialysis mode (none, hemodialysis, or peritoneal dialysis), and its duration prior to transplantation were also recorded. HLA mismatches were recorded for HLA-A, -B, and -DR loci. Other relevant immunological parameters such as, pre-transplant panel reactive antibodies (0 vs. positive at any level), and transplant type (living/deceased) were analyzed as covariates. Cold ischemia time, donor age, and presence of delayed graft function were also considered. Methods of assessment and definitions of these variables have been previously described in details (6).

### Flow cytometry of whole blood

Peripheral blood mononuclear cells were isolated by density gradient centrifugation (Pancoll; Pan-Biotech GmBH, Aidenbach, Germany). Cells were stained with the following conjugated antibodies directed against CD3, CD4, CD8, CD31, CD45RA CD16, CD56, CD14 and CD45RO. The identification of lymphocyte subpopulations is carried out using four combinations of cluster of differentiation. The source and clone of each antibody is specified in the **Supplementary materials and methods**. Immune markers used in PCA included lymphocytes, monocytes and Natural Killer (NK) cells identified by: T cells (CD3+), CD4+ T cells (CD3+CD4+CD8-), CD8+ T cells (CD3+CD8+CD4-), B cells (CD19+), NK cells (CD56+CD3-), naive CD4+ T cells (CD4+CD45RA+), memory CD4+ T cells (CD4+CD45RO+), RTE (CD4+CD45RA+CD31+), classical monocytes (CD14+CD16-) and intermediate monocytes (CD14+CD16+). Antibody clones and gating strategies are detailed in **Supplementary materials and methods**. For exploring the association between lymphocytes subsets and acute rejection, CD45RA and CCR7 serve as defining four T cell subsets (naïve, CD45RA+CCR7+; effector memory, CD45RA-CCR7-; central memory, CD45RA-CCR7+; effector memory expressing CD45RA, CD45RA+CCR7-) (11).

Cell debris and doublets were excluded on the basis of side versus forward scatter. Percentage of T cells, B cells, NK cells and monocytes were determined on fresh samples by flow cytometry with an FC500 cytometer (Beckman Coulter, Villepinte, France) according to the manufacturer’s recommendations. Absolute numbers of different immune population, CD4+ and CD8+ T cells were determined on fresh samples by a single platform flow cytometry approach using the TetraCXP method, Flow-Count fluorospheres, and the same cytometer.

### Outcomes

Acute rejection was considered in the presence of serum creatinine elevation. Only biopsy-proven acute rejections were considered. Acute rejection was defined according to the Banff classification (12). Only cellular acute rejections were considered.

Delayed Graft Function (DGF) was considered when dialysis is needed in the first week after transplantation.

### Multiparameter analysis and hierarchical clustering

Pre-transplant immunological populations were used for the Principal Component Analysis (PCA) (CD3+, CD3+CD4+, CD45RO+CD4+, CD45RA+CD4+, CD31+CD45RA+CD4+, CD3+CD8+, CD19+, CD3+CD56+, CD45CD14+, CD45CD14+DR+ cell count and frequency as well as CD4/CD8 ratio). Only patients with all available analyses were included. We retained components with eigenvalues above 1. We then performed unsupervised hierarchical clustering based on the significant components using Ward’s method with Euclidian distances. The number of clusters was selected based on the higher relative loss of inertia criteria [iclusters n+1/i(cluster n)]. Detailed analysis is depicted in **Supplementary materials and methodes**. PCA and clustering were performed using the FactoMineR version 2.3 package with R version 4.0.2 (R Core Team, Vienna, Austria).

### Statistical analyses

Clinical characteristics of the participants were described with mean expressed as +/- SD, median with the interquartile range (IQR) and numbers of events with percentage. After PCA and clustering, each cluster was described using the 18 quantitative immunologic parameters as well as clinical characteristics. Cluster comparisons were based on analysis of variance (ANOVA) for quantitative variables and chi-2 test for categorical variables (or Fischer test when appropriate).

Survival without acute rejection analysis was then performed for the clusters using Kaplan-Meier survival curves. As patient may die or return to dialysis before experiencing the outcome of interest, death from any cause as well as graft failure were considered as competing risks. Therefore, we used a competing-risk approach. The time-to-event was calculated from the date of transplantation to the outcome (acute rejection). Cumulative incidence function curves were also constructed for each cluster. The Fine-Gray model was used to analyze the prognostic effect of belonging to a specific cluster on the sub-distribution hazard function of the outcome (13). Unadjusted as well as age- and gender-adjusted sub-distribution relative hazards were estimated with the corresponding 95% confidence intervals (CI). Competing risk analysis was performed with R using the survival and prodlim version 2019.11.13 packages or using Prism, version 5.0 software (GraphPad Software, San Diego, CA) and SAS software (SAS institute Inc., Cary, NC).

## Results

### Study population

Characteristics of the study population were depicted in **Table 1**. Mean age was 53 ± 14 years and about two thirds of patients were male. Twenty percent had diabetes mellitus. All the patients had at least a one-year follow-up. The rate of missing data was < 5 percent for all studied parameters.

**Table 1:** Clinical characteristics of the study population.

Characteristics	Overall patients (No. = 832)
Age, year, mean (SD)	53 (14)
Median (IQR)	54 (44-63)
Male gender, n (%)	515 (62%)
BMI, kg/m², mean ± SD	26 (5)
Median (IQR)	25 (22-29)
* Missing*	*19*
Hypertension, n (%)	
No	119 (15%)
Yes	694 (85%)
* Missing*	*19*
Diabetes, n (%)	
No	659 (80%)
Yes	161 (20%)
Missing	12
AntiHLA immunization, n (%)	232 (28%)
Dialysis antecedent, n (%)	740 (90%)
* Missing*	*11*
Hemodialysis, n (%)	601 (81%)
Peritoneal dialysis, n (%)	139 (19%)
Causal Nephropathy	
Glomerulopathy	170 (21%)
Vascular nephropathy	79 (10%)
Chronic interstitial nephropathy	48 (6%)
Congenital	12 (1%)
Polycystic	139 (17%)
Diabetes	99 (12%)
Other	258 (32%)
* Missing*	*27*
Anti-CMV antibodies, n (%)	
+	471 (57%)
-	352 (43%)
* Missing*	*9*
Induction therapy, n (%)	
No	76 (9%)
aCD25mAb	528 (65%)
ATG	205 (26%)
* Missing*	*23*

ATG, antithymocyte globulin; CMV, cytomegalovirus; aCD25mAb, anti CD25 monoclonal antibody; HLA, human leucocyte antigen.

### Identification of distinct patient groups using hierarchical clustering of immune profiles

**Figure 2:**
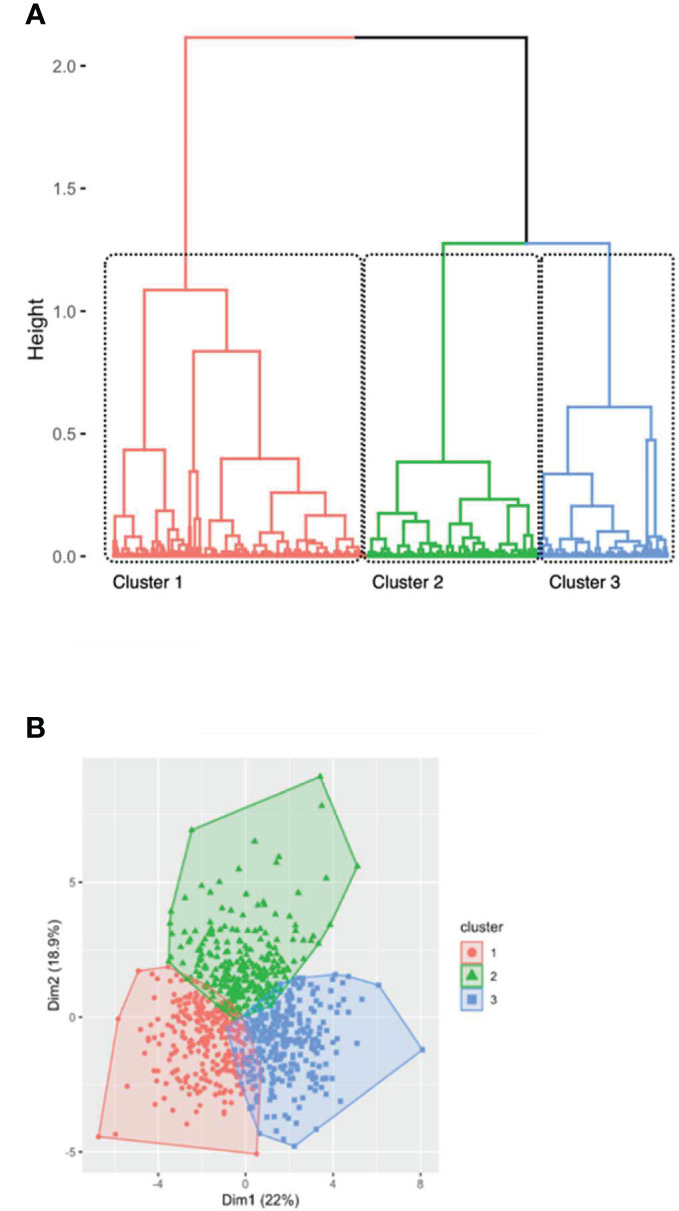
Hierarchical clustering of patients after principal component analysis **(A)** represented by the dendrogram of patients and **(B)** by a scatter view plot. **(A)** identification of 3 clusters among 832 first transplant recipients according to immunological data. Profiles were assigned based on the separation of the clustering trees. **(B)** Colors were based on clustering profile and mainly defined by dimension 2 and dimension 1 in our hierarchical clustering. Three clusters were identified: older immunity in red (cluster 1), activated immunity in green (cluster 2) and naïve immunity in blue (cluster 3).

A PCA was conducted on immune cells (frequency and total amount). PCA identified three clusters of individuals based on the distances between each branches of the dendogram (**Figure 2A**). Clusters, determined by the unsupervised hierarchical clustering analysis, were supposed to define immune profile and reflect groups of patients with similar immunophenotype. Three clusters were isolated (**Figure 2B**; **Table 2**).

**Table 2:** Immune population phenotype at the day of transplantation among the different clusters determined by hierarchical clustering.

	Memory immunity (cluster 1, n=271)	Activated immunity (cluster 2, n=270)	Naïve immunity (cluster 3, n=291)	*p* value
Tcell (CD3+) (n/mm3)	732 ( ± 285)	1337 ( ± 466)	939 ( ± 374)	<0.001
Tcell (CD3+) (%)	68.1 ( ± 9.4)	80.6 ( ± 5.8)	80.1 ( ± 6.3)	<0.001
CD4+ Tcell (CD3+CD4+) (n/mm3)	465 ( ± 202)	773 ( ± 320)	702 ( ± 302)	<0.001
CD4+ Tcell (CD3+CD4+) (%)	42.8 ( ± 8.2)	45.9 ( ± 7.6)	59.6 ( ± 6.9)	<0.001
CD8+ Tcell (CD3+CD8+) (n/mm3)	248 ( ± 123)	531 ( ± 205)	218 ( ± 97)	<0.001
CD8+ Tcell (CD3+CD8+) (%)	23.4 ( ± 7.9)	32.7 ( ± 8.1)	18.9 ( ± 6.0)	<0.001
CD4+/CD8+ ratio	2.1 ( ± 0.9)	1.5 ( ± 0.6)	3.6 ( ± 1.5)	<0.001
Bcell (CD19+) (n/mm3)	111 ( ± 93)	161 ( ± 199)	90 ( ± 64)	<0.001
Bcell (CD19+) (%)	10.1 ( ± 6.2)	8.9 ( ± 6.6)	7.4 ( ± 3.9)	<0.001
Naïve CD4+ Tcell (CD3+CD4+CD45RA+) (n/mm3)	29.5 ( ± 12.3)	36.6 ( ± 13.5)	50.2 ( ± 12.0)	<0.001
Memory CD4+ Tcell (CD3+CD4+CD45RO+) (n/mm3)	70.2 ( ± 12.5)	63.4 ( ± 13.5)	49.6 ( ± 12.2)	<0.001
RTE (CD3+CD4+CD45RA+CD31+) (n/mm3)	18.3 ( ± 9.0)	23.9 ( ± 11.5)	32.3 ( ± 10.7)	<0.001
NK cell (CD56+) (n/mm3)	223.1 ( ± 135.2)	167.3 ( ± 126.7)	130.5 ( ± 82.4)	<0.001
NK cell (CD56+) (%)	20 ( ± 9)	10 ( ± 4)	11 ( ± 6)	<0.001
Monocyte (CD14+) (n/mm3)	456 ( ± 277)	400 ( ± 162)	403 ( ± 210)	0.004
Monocyte (CD14+) (%)	7.9 ( ± 3.4)	6.2 ( ± 2.3)	7.0 ( ± 2.6)	<0.001
Infl. Monocyte (CD14+CD16+) (n/mm3)	62 ( ± 61)	45 ( ± 39)	46 ( ± 38)	<0.001
Infl. Monocyte (CD14+CD16+) (%)	1.2 ( ± 1.6)	0.7 ( ± 0.6)	0.8 ( ± 0.6)	<0.001

RTE, recent thymic emigrant - ANOVA for quantitative variable.

A first cluster (n=271 patients) was characterized by an increase in memory CD4+ T cells and a decrease in naive CD4+ T cells (including central CD4+ T cells). However, the absolute number of immune cell (representing by T cell count) was low. This cluster was called “memory immune profile”. A second cluster (n=270 patients) was characterized by an increased number of immune cells especially CD8+ T cells (in percentage and absolute number). The total rate of T cells was increased but the CD4/CD8 ratio was lower suggesting an “activated immune profile”. A third cluster (n=291 patients) was characterized by increased naive immune cells, mostly naive CD4+ T cells and central CD4+ T cells defining a “naïve immune profile”. Of note, all cell subsets counts and frequencies differed in all three groups (**Table 2**).

### Immune cell profiles correlate with clinical phenotypes

We analyzed and compared demographics and clinical characteristics of the three immune profiles. The results are summarized in **Table 3**.

**Table 3:** Clinical characteristics among the 3 immune profiles determined with hierarchical clustering.

Characteristics	Memory immunity (cluster 1, n=271)	Activated immunity (cluster 2, n=270)	Naïve immunity (cluster 3, n=291)	*p* value
Age, year, mean (SD)	56 ( ± 13)	48 ( ± 15)	54 ( ± 12)	<0.001
Median (IQR)	58 (48 - 67)	47 (36 - 60)	55 (47 - 63)	
Male gender, n (%)	183 (68%)	162 (60%)	170 (58%)	0.098
BMI, kg/m², mean ± SD	27 ( ± 5)	25 ( ± 5)	26 ( ± 5)	0.004
Median (IQR)	26 (23 - 30)	25 (22 - 28)	25 (22 - 29)	
Missing	8	4	7	
Hypertension, n (%)				0.110
No	29 (11%)	41 (15%)	49 (17%)	
Yes	234 (86%)	226 (84%)	234 (80%)	
Missing	8 (3%)	3 (1%)	8 (3%)	
Diabetes, n (%)				0.003
No	196 (72%)	219 (81%)	244 (84%)	
Yes	73 (27%)	49 (18%)	39 (13%)	
Missing	2 (1%)	2 (1%)	8 (3%)	
Anti-HLA immunization, n (%)				0.082
No	197 (73%)	176 (65%)	197 (68%)	
Yes	65 (24%)	89 (33%)	78 (27%)	
Dialysis antecedent, n (%)				0.005
No	15 (6%)	41 (15%)	25 (9%)	
Hemodialysis, n (%)	201 (74%)	168 (62%)	192 (66%)	
Peritoneal dialysis, n (%)	40 (15%)	46 (17%)	53 (18%)	
DP/HD	14 (5%)	13 (5%)	13 (4%)	
Missing	1 (0%)	1 (0%)	5 (2%)	
Causal Nephropathy				<0.001
Glomerulopathy	53 (20%)	61 (23%)	56 (19%)	
Vascular nephropathy	29 (11%)	23 (9%)	27 (9%)	
Chronic interstitial nephropathy	17 (6%)	16 (6%)	15 (5%)	
Congenital	4 (1%)	5 (2%)	3 (1%)	
Polycystic	35 (13%)	26 (10%)	78 (27%)	
Diabetes	52 (19%)	25 (9%)	22 (8%)	
Other	49 (18%)	68 (25%)	57 (20%)	
unspecified	23 (8%)	40 (15%)	21 (7%)	
Missing	9 (3%)	6 (2%)	12 (4%)	
Anti-CMV antibodies, n (%)				<0.001
+	152 (56%)	205 (76%)	114 (39%)	
-	116 (43%)	64 (24%)	172 (59%)	
Missing	3 (1%)	1 (0%)	5 (2%)	

Fisher test for binary variable, Chi2 for more than 2 modality variable, ANOVA for quantitative variable.

Patients classified as having a memory immune profile were older, had higher BMI, and were more prone to have type 2 diabetes. Patients assigned to the activated immune profile were younger and were more likely to have had pre-transplant CMV exposure. Patients allocated to the naïve immune profile were more frequently CMV-naïve. Other characteristics did not differ between the three immune profiles. Consequently, main demographic and clinical drivers of pre-transplant immune status were age, CMV status, and diabetes.

### Immune profiles are associated with acute rejection

**Figure 3:**
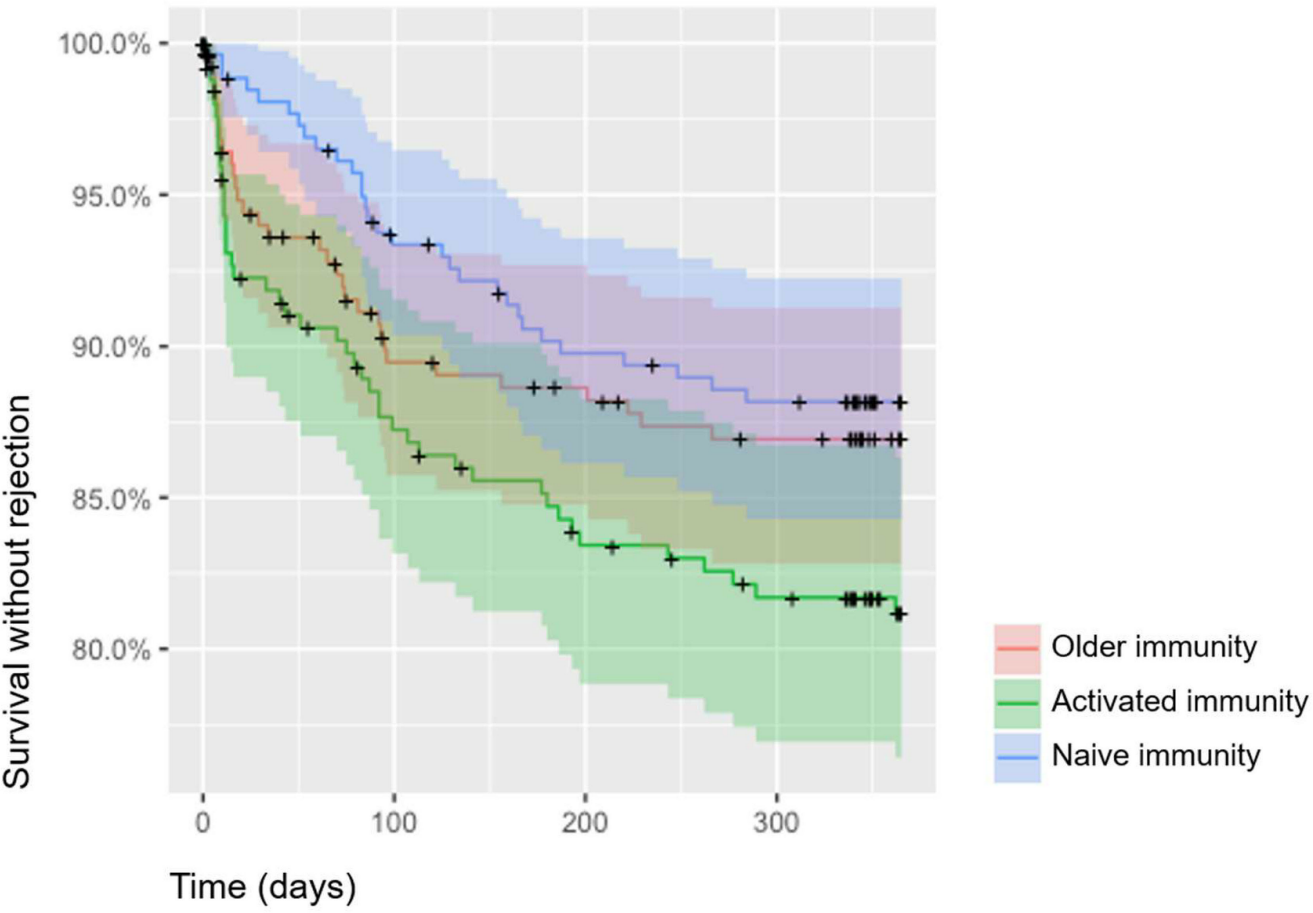
Kaplan Meier curves for survival without acute rejection according to cluster belinging. Three clusters were identified: memory immunity in red (cluster 1), activated immunity in green (cluster 2) and naïve immunity in blue (cluster 3).

**Figure 4:**
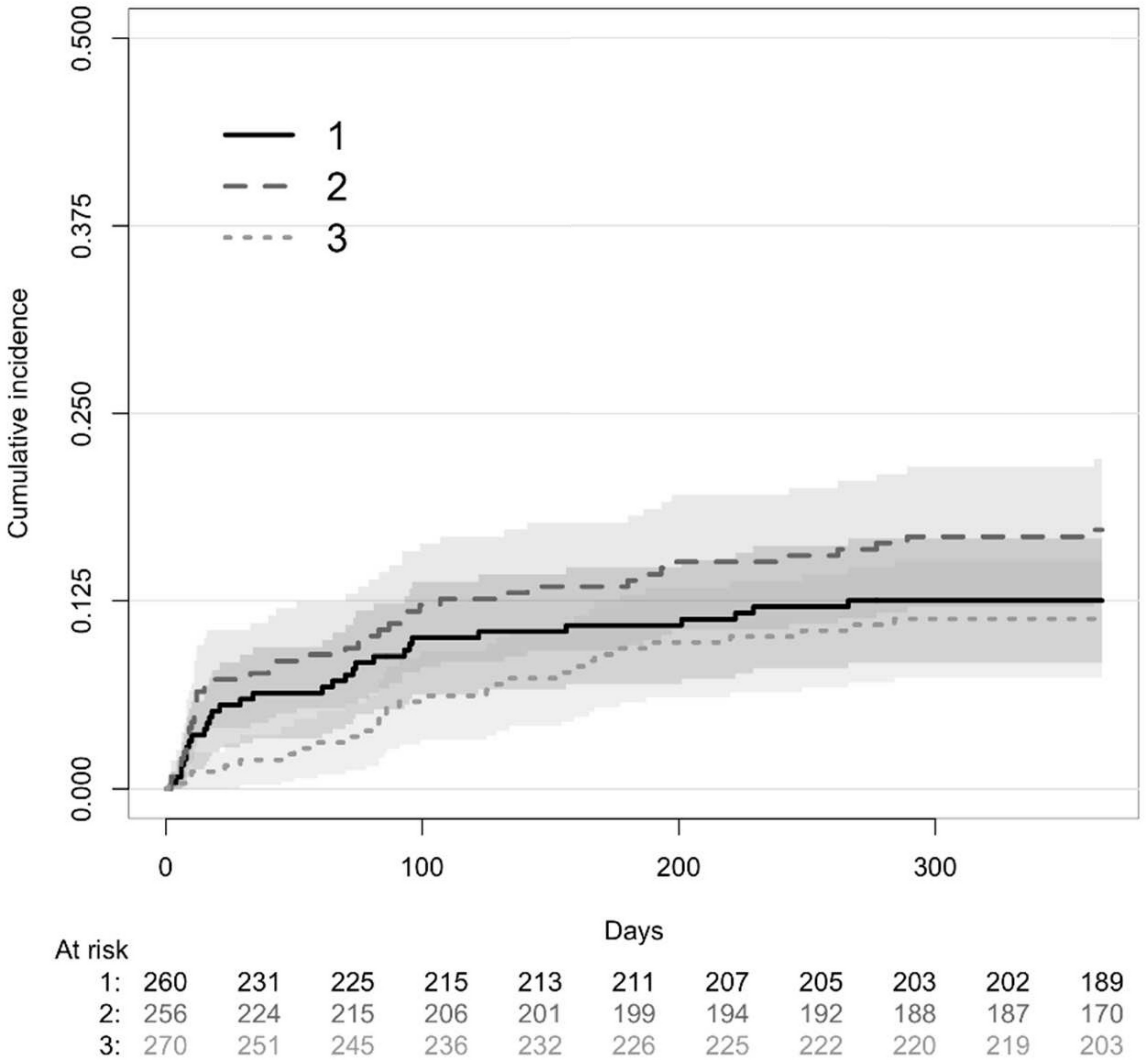
Competitive risk of death and acute rejection at 1 years post transplantation according to clusters determined with principal component analysis identified as older immunity (cluster 1), activated immunity (cluster 2) and naïve immunity (cluster 3). Three clusters were identified: memory immunity with full line (cluster 1), activated immunity with dashed line (cluster 2) and naïve immunity with dotted line (cluster 3).

There was no difference in induction therapy or maintenance immunosuppressive treatments between the 3 immune profiles (**Table 3**). The proportion of patients with DGF was also similar in the 3 groups. One hundred and seven patients (13.6%) experienced acute rejection in the first year post-transplant. Mean time between transplantation and acute rejection was 105 +/- 88 days. Patients exhibiting an activated immune profile were more prone to develop acute rejection that both those with a naïve immune profile (HR=1.68, IC95% [1.06; 2.67], p=0.027) and those with a memory immune profile (HR 1.32, IC95% [0.95-1.88], p=0.096) (**Figure 3**). A competitive analysis taken death into account provided similar results (HR=1.606, 95%IC [1.00-2.58], p=0.0498) (**Figure 4**).

Because activated immune profile is mainly defined by CD8+ T cell expansion, we analyzed whether CD8+ T cell count and/or frequency may replicate previous results. Neither CD8+ T cell count nor frequency were associated with the occurrence of acute rejection (**Supplementary data**, **Tables S1A, B**). Patients were divided into three groups corresponding to tertiles of CD8+ T cells count: 39 - 217 CD8+/mm3, 217 - 374 CD8+/mm3, 374 - 1400 CD8+/mm3. Frequency of acute rejection was similar in the three tertiles of CD8+ T cells (**Supplementary data**, **Figure S1A**). There was also no difference according to the percentage of CD8+ T cells (**Supplementary data**, **Figure S1B**). No other T cell subset was associated with acute rejection. ATG profoundly affects lymphocyte counts and phenotype. We separately studied the association between clusters and acute rejection in patients having or not received ATG. The association between clusters and acute rejection was similar in both groups of patients.

Delayed graft function (HR=1.48, IC95% [1.01-2.18], p=0.046) was also associated with acute rejection and retained in the multivariate analysis. Age, CMV status, and diabetes, closely linked to cluster definitions, were forced into the model. After multivariate analysis, the activated immune profile was still associated with acute rejection (HR=1.69, IC95% [1.05-2.70], p=0.030 and HR=1.85, IC95% [1.16; 3.00], p=0.011, versus the “memory immune profile” and the “naïve immune profile”, respectively). DGF (HR=1.50, IC95% [1.02-2.33], p=0.040) remained associated with acute rejection. Age, gender, and diabetes mellitus were not associated with acute rejection.

### Subsequent analyses of the activated immune profile

**Figure 5:**
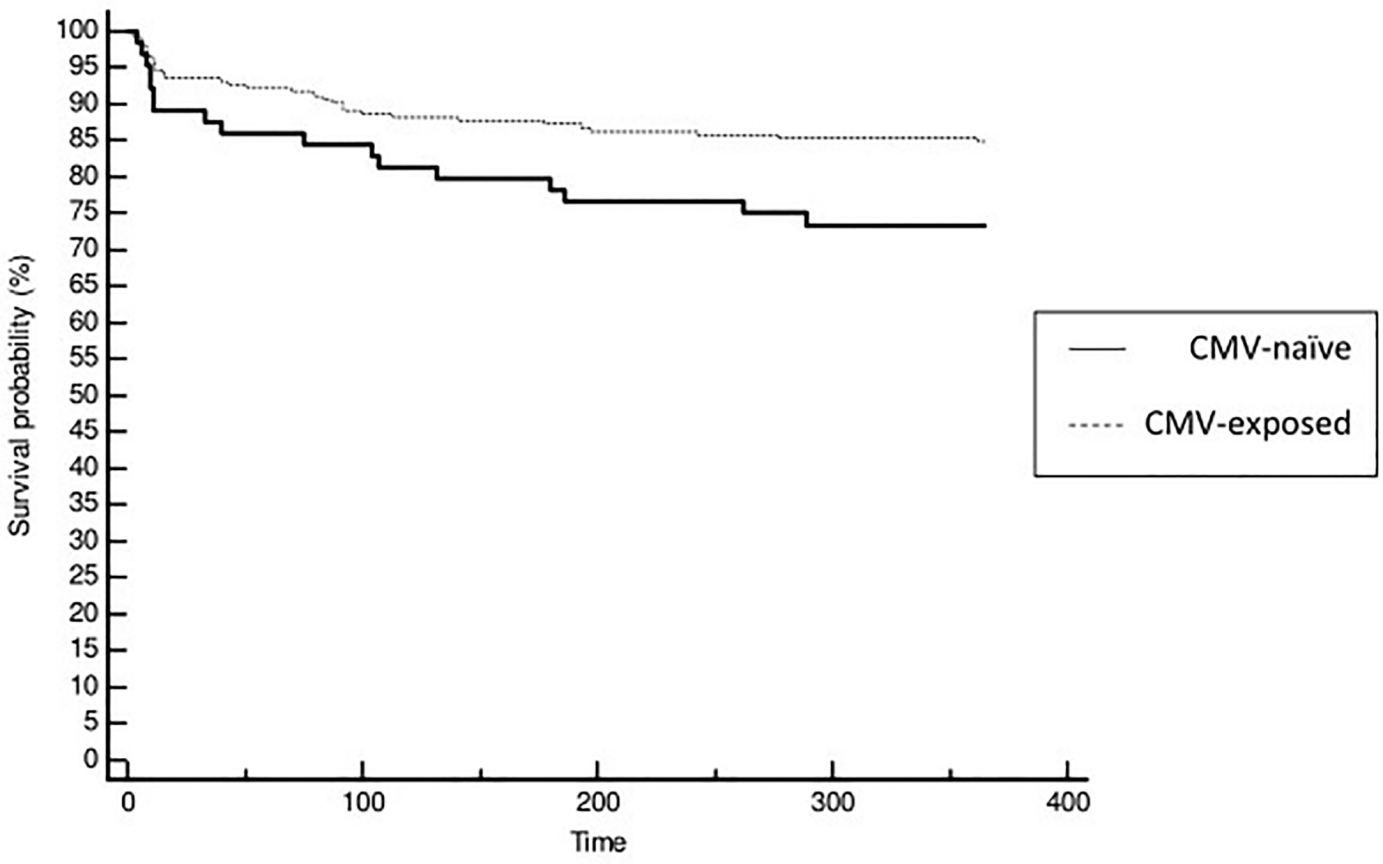
Kaplan Meier curves for survival without acute rejection according to patients CMV status of cluster 2 (activated immunity). Patients naïve to CMV exposure were identified with full line and patient exposed to CMV with dashed line.

The activated immune profile shares some similarities with the immune risk profile, which is mainly characterized by CD8+ T cell expansion and CMV seropositivity. Nevertheless, 24% of patients assigned to the activated immune profile were CMV-naïve (**Table 3**). CMV-exposed patients tended to be older (49+/-14 vs 45+/-15 years, p=0.087). Although CMV-exposed patients had moderately higher CD8 T cell count than CMV-naïve patients, they had similar CD8 frequency (37 + 11 vs 39 + 11, p=0.399). We observed that acute rejection was less frequent in CMV-exposed patients (16 vs 27%, HR=0.54, IC95% [0.30; 0.97], p=0.039) (**Figure 5**; **Supplementary data**, **Table S2**).

We therefore hypothesized that CD8 subset distribution could be different between CMV-naïve and CMV-exposed patients. Naïve CD8+ T cell (CD8+CD45RA+CCR7+) frequency was much higher (35 + 18 vs 27 + 18%, p=0.006) in CMV-naïve patients, whereas frequency of TEMRA CD8 T cells (CD8+CDRA+CCR7-) tended to be lower (42 + 18 vs 47 + 16%, p=0.059) (**Supplementary data**, **Table S2**). Nevertheless, none of these subsets was associated with acute rejection.

## Discussion

We report in a large cohort of kidney transplant recipients that immune profile determination based on PCA defines clinically different sub-groups of population and may help to discriminate patients at-risk for acute rejection. The association between immune profiles and acute rejection was independent and observed in all sub-groups of patients. A specific category of patients with high CD8+ T cells level and no past exposure to CMV seems to be at the highest risk of acute rejection. Using a large panel of immune cellular subtypes, we isolated three immune profiles with distinct clinical phenotypes. Clinical phenotypes were concordant with expected immune profiles. Three main clinical characteristics seems to drive immune clustering, namely age, diabetes, and CMV exposure. Immune senescence is driven by ageing and some studies report increased senescence in patients with insulin resistance (14, 15). Persistent CMV infection leads to chronic stimulation of CD8 T cells, which expand clonally exhibiting an effector memory phenotype. Meijers et al. showed that TEMRA CD8^+^ T cell expansion in end-stage renal disease patients were highly associated with CMV exposure (16). Clinical correlation may be considered as an internal validation for the relevance of the immune clusters defined by PCA.

The “activated immune profile” shares some similarities with the immune risk phenotype (IRP). IRP was first defined as the association of high CD8 and low CD4 numbers, and poor proliferative response to concanavalin A (17). Further studies suggested that IRP could be defined using only the inverted CD4/CD8 ratio (18). Further studies have extended these results (19, 20). CMV exposure and an increase in the number of lately differentiated CD8+ CD28 effector cells have been linked to IRP. More recently, IRP was also found to be more prevalent in younger CMV-exposed patients (21). The “activated immune profile” is mainly characterized by high CD8+ T cells count, lower CD4/CD8, and CMV seropositivity, which are predominant features of the IRP. Nevertheless, the “activated immune profile” is likely to be different from IRP. Patients with IRP have typically low CD4+ T cells whereas this subset is high in patients with “activated immune profile”. Moreover, most patients do not have inverted CD4/CD8 ratio. Finally, one quarter of patients in the activated immune profile were CMV-naïve.

Distributions of both CD4+ and CD8+ T cells profiles are largely different between CMV-naïve and CMV-exposed patients and suggest more pronounced T cell exhaustion in CMV-positive patients. However, CMV-naïve patients assigned to the activated immune profile appear to be at very high risk of acute rejection. Consistent with this result, Betjes et al. reported that expansion of terminally differentiated CD8+ TEMRA protects against acute rejection after kidney transplantation (22). Consistent with this result, we also showed a trend towards a lower incidence of acute rejection in patients with pre-transplant IRP (23). Nevertheless, the role of TEMRA in acute rejection is probably more complex as recent studies identified this population as being associated with both acute rejection and graft loss (24, 25).

However, in our study, no specific T cell subset was associated with acute rejection.

Indeed, despite subsequent phenotyping of T cells, we were unable to identify a specific subset explaining the association between having “an activated immune profile” and an increased incidence of acute rejection. We assume that this point reinforces our primary hypothesis. It suggests that a combination of different immune actors and multiple interactions rather than a unique factor contributes to acute rejection. We now have to integrate the complexity of immune interactions in our predicting models.

It is possible to plot supplementary individuals onto the principal axes. Using the formulae allowing principal components computations, we simply have to compute linear combinations of these supplementary point characteristics. Thus, a next patient may be included in a defined cluster before, subject to determination of immune parameters. This offers the opportunity of external validation of the PCA. Finally, clinical use may be generalized to classify patients and predict their risk category regarding acute rejection.

Our study has some limitations. Even when acute rejection was clearly defined, there was no centralized analysis of graft histology. Nevertheless, misclassification remains unlikely. The indication for biopsy may have varied from one center to another, but without any possible relationship with PCA. Importantly, systematic biopsies were not considered and only for cause-biopsies were analyzed. Due to the prospective design of the study, the rate of missing data was very low (<5%). Underreporting of events is unlikely in the early post-transplant period when all the patients are still followed in the transplant center.

Using PCA, we defined a subset of kidney transplant recipients carrying a high risk of acute rejection. These patients are characterized by an “activated immune profile” in the absence of previous exposure to CMV. Global approach of immune equilibrium may be more relevant to predict immune-related events than analysis of a single parameter.

## Data availability statement

The raw data supporting the conclusions of this article will be made available by the authors, without undue reservation.

## Ethics statement

The studies involving human participants were reviewed and approved by the ethics committee of the Franche-Comté study. The patients/participants provided their written informed consent to participate in this study.

## Author contributions

EG and DD are the guarantors. DD, EG, MC, MD, and PS designed the study. CL, MG, ND, and SB carried out experiments. CC, CL, DD, EG, FL, JB, MC, ND, and PS supported (administrative, technical or material) the study. DD, EG, and PS supervised the study. CL, DD, EG, FL, MC, MD, MG, ND, and SB acquired, analyzed or interpreted the data. DD, EG, MC, and MD drafted the manuscript; DD, EG, MD, ND, and PS revised the paper; DD and PS obtained funding. All authors contributed to the article and approved the submitted version.
