# Supplementary material for: Pre-transplant immune profile defined by principal component analysis predicts acute rejection after kidney transplantation
Source: Front Immunol. 2023 Jul 11;14:1192440. doi: 10.3389/fimmu.2023.1192440 (PMC10367005; doi:10.3389/fimmu.2023.1192440)
Supplement: Supplementary file 1 [file DataSheet_1.docx]

**Pre-transplant immune profile defined by principal component analysis predicts acute rejection after kidney transplantation**

Emilie Gaiffe^1, 2^, Mathilde Colladant, Maxime Desmarets, Jamal Bamoulid, Franck Leroux, Caroline Laheurte, Sophie Brouard^3^, Magali Giral^3^, Philippe Saas, Cécile Courivaud, Nicolas Degauque^3^ and Didier Ducloux.

**Supplementary materials and methods**

**Antibody clones**

The identification of the lymphocyte subpopulations is carried out using 4 combinations of cluster of differentiation (CD) described in the table below. The source (BC, Beckman Coulter, BD, BD Biosciences) and clone of each antibody are specified for each combination.

T cells (CD3+)

CD4+ T cells (CD3+CD4+CD8-)

CD8+ T cells (CD3+CD8+CD4-)

| **Conbination** | **CD** | **Fluorochrome** | **Clone** | **Reference** | **Firme** | **Volume** |
| --- | --- | --- | --- | --- | --- | --- |
| 1 | CD3 | FITC | UCHT1 | A07746 | BC | 10µL |
|  | CD8 | PE | B9.11 | A07757 | BC | 10µL |
|  | CD16 | PC7 | 3G8 | 6607118 | BC | 5µL |
|  | CD19 | APC | J3-119 | IM2470 | BC | 5µL |
| 2 | CD45RA | FITC | HI100 | 555488 | BD | 10µL |
|  | CD31 | PE | 1F11 | IM2409 | BC | 10µL |
|  | CD45RO | ECD | UCHL1 | B49192 | BC | 10µL |
|  | CD4 | APC | RPA-T4 | 555349 | BD | 5µL |
| 3 | HLA DR | FITC | G46-6 | 555811 | BD | 10µL |
|  | CD86 | PE | HA5.2B7 | IM2729U | BC | 10µL |
|  | CD14 | ECD | RMO52 | B92391 | BC | 10µL |
|  | CD16 | PC7 | 3G8 | 6607118 | BC | 5µL |
|  | CD45 | APC | HI30 | 555485 | BD | 5µL |
| 4 | CD56 | PC7 | N901 | A21692 | BC | 5µL |
|  | CD3 | PC5 | UCHT1 | 6607013 | BC | 10µL (Tetrachrome) |
|  | CD8 | ECD | SFCI21Thy2D3 | 6607013 | BC |  |
|  | CD4 | RD1 (PE) | SFCI12T4D11 | 6607013 | BC |  |
|  | CD45 | FITC | B3821F4A | 6607013 | BC |  |

**Cytometry gating strategies**

The gating strategy is carried out as presented below (examples of gating).

**
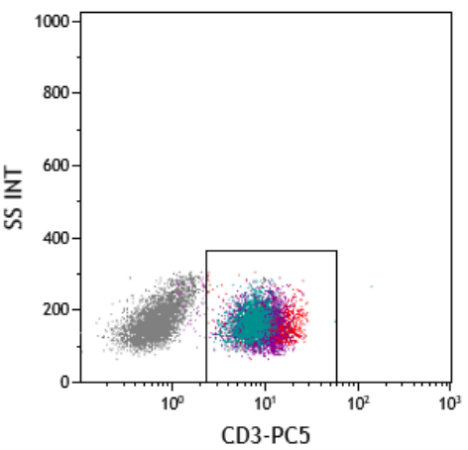
**

*T cells (CD3+) CD4+ T cells (CD3+CD4+CD8-)*

*CD8+ T cells (CD3+CD8+CD4-)*

**
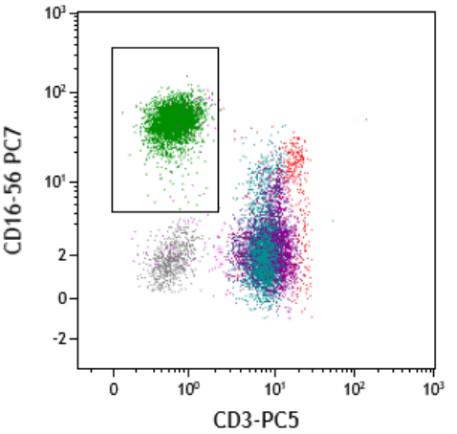

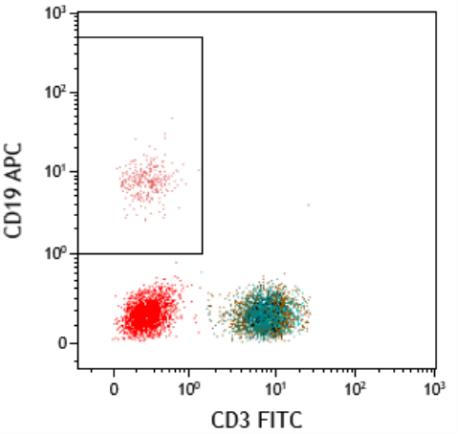
**

*B cells (CD19+)* *NK cells (CD56+CD3-)*

**
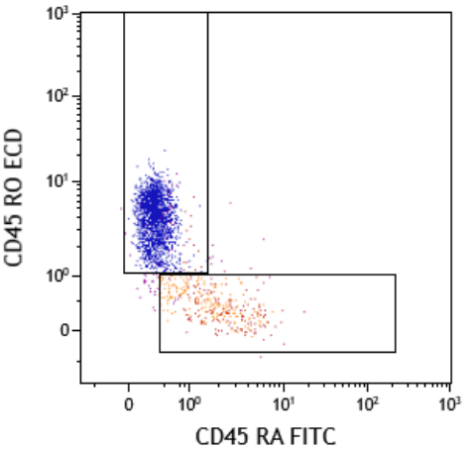
**

*Naïve CD4+ T cells (CD4+CD45RA+) Central CD4+ T cells*

*Memory CD4+ T cells (CD4+CD45RO+) (CD4+CD45RA+CD31+)*

**
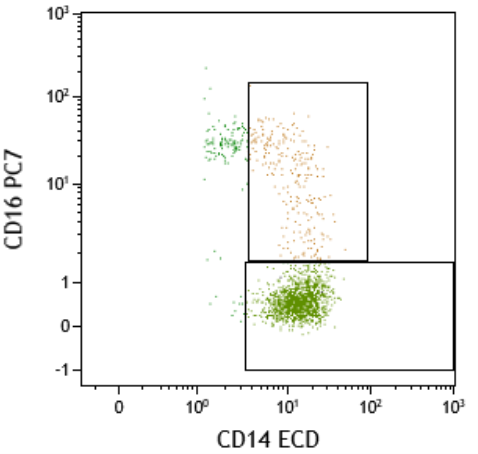
**

*Classical monocytes (CD14+CD16-)*

*Intermediate monocytes (CD14+CD16+)*

**Clustering and ACP detailed description**

The eigenvalues of each component are described below:

| Composante | EIngenvalue | Percentage of variance | Cumulative percentage of variance |
| --- | --- | --- | --- |
| 1 | 3.96 | 22.1 | 22.1 |
| 2 | 3.41 | 18.95 | 40.96 |
| 3 | 2.97 | 16.48 | 57.43 |
| 4 | 2.29 | 12.73 | 70.16 |
| 5 | 1.64 | 9.13 | 79.29 |
| 6 | 1.48 | 8.23 | 87.52 |
| 7 | 0.0.81 | 4.53 | 92.05 |
| 8 | 0.44 | 2.46 | 94.51 |
| 9 | 0.22 | 1.23 | 95.75 |
| 10 | 0.2 | 1.11 | 96.86 |
| 11 | 0.17 | 0.96 | 97.82 |
| 12 | 0.14 | 0.80 | 98.62 |
| 13 | 0.12 | 0.65 | 99.27 |
| 14 | 0.05 | 0.30 | 99.56 |
| 15 | 0.04 | 0.23 | 99.80 |
| 16 | 0.02 | 0.13 | 99.92 |
| 17 | 0.01 | 0.07 | 100.00 |
| 18 | 0.00 | 0.00 | 100.00 |


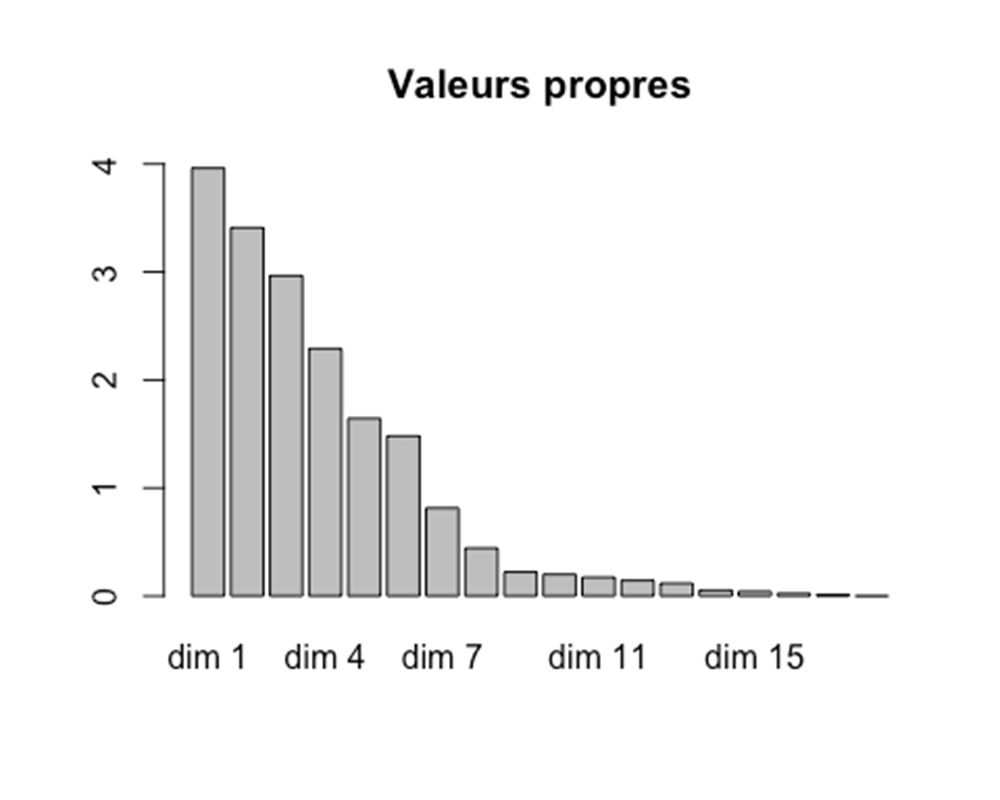


Detailed description of each retained dimension (eigenvalue >1; cor, correlation ; p, p value)

| **Immunological parameter** | **D1** | | **D2** | | **D3** | | **D4** | | **D5** | | **D6** | |
| --- | --- | --- | --- | --- | --- | --- | --- | --- | --- | --- | --- | --- |
|  | **cor** | **p** | **cor** | **p** | **cor** | **p** | **cor** | **p** | **cor** | **p** | **cor** | **p** |
| **n T cells (CD3+)** | **0.379** | **<0.0001** | **0.729** | **<0.0001** | **0.479** | **<0.0001** |  |  |  |  |  |  |
| **% T cells (CD3+)** | **0.534** | **<0.0001** | **0.457** | **<0.0001** | **-0.437** | **<0.0001** | **0.504** | **<0.0001** |  |  | **-0.276** | **<0.0001** |
| **n CD4+ T cells (CD3+CD4+CD8-)** | **0.561** | **<0.0001** | **0.448** | **<0.0001** | **0.540** | **<0.0001** |  |  | **-0.176** | **<0.0001** | **-0.349** | **<0.0001** |
| **% CD4+ T cells (CD3+CD4+CD8-)** | **0.782** | **<0.0001** | **-0.274** | **<0.0001** | **0.294** | **<0.0001** |  |  | **-0.367** | **<0.0001** | **-0.208** | **<0.0001** |
| **n CD8+ T cells (CD3+CD8+CD4-)** |  |  | **0.913** | **<0.0001** | **0.236** | **<0.0001** |  |  | **0.208** | **<0.0001** |  |  |
| **% CD8+ T cells (CD3+CD8+CD4-)** | **- 0.350** | **<0.0001** | **0.753** | **<0.0001** | **-0.328** | **<0.0001** | **0.171** | **<0.0001** | **0.318** | **<0.0001** | **0.165** | **<0.0001** |
| **R CD4/CD8** | **0.544** | **<0.0001** | **-0.621** | **<0.0001** | **0.193** | **<0.0001** |  |  | **-0.350** | **<0.0001** | **-0.218** | **<0.0001** |
| **% naive CD4+ T cells (CD4+CD45RA+)** | **0.808** | **<0.0001** | **-0.153** | **<0.0001** |  |  | **-0.234** | **<0.0001** | **0.418** | **<0.0001** | **0.231** | **<0.0001** |
| **% central CD4+T cells (CD4+CD45RA+CD31+)** | **0.732** | **<0.0001** |  |  |  |  | **-0.293** | **<0.0001** | **0.409** | **<0.0001** | **0.268** | **<0.0001** |
| **% memory CD4+ T cells (CD4+CD45RO+)** | **-0.808** | **<0.0001** | **0.160** | **<0.0001** |  |  | **0.234** | **<0.0001** | **-0.419** | **<0.0001** | **-0.226** | **<0.0001** |
| **n B cells (CD19+)** |  |  | **0.367** | **<0.0001** | **0.567** | **<0.0001** | **-0.372** | **<0.0001** | **-0.420** | **<0.0001** | **0.382** | **<0.0001** |
| **% B cells (CD19+)** | **-0.135** | **<0.0001** |  |  | **0.411** | **<0.0001** | **-0.431** | **<0.0001** | **-0.470** | **<0.0001** | **0.606** | **<0.0001** |
| **n NK cells (CD56+CD3-)** | **-0.213** | **<0.0001** |  |  | **0.671** | **<0.0001** | **-0.327** | **<0.0001** | **0.304** | **<0.0001** | **-0.453** | **<0.0001** |
| **% NK cells (CD56+CD3-)** | **-0.494** | **<0.0001** | **-0.523** | **<0.0001** | **0.332** | **<0.0001** | **-0.360** | **<0.0001** | **0.326** | **<0.0001** | **-0.283** | **<0.0001** |
| **n classical monocytes (CD14+CD16-)** |  |  |  |  | **0.650** | **<0.0001** | **0.488** | **<0.0001** |  |  |  |  |
| **% classical monocytes (CD14+CD16-)** | **-0.132** | **0.0001** | **-0.295** | **<0.0001** | **0.351** | **<0.0001** | **0.614** | **<0.0001** |  |  | **0.217** | **<0.0001** |
| **n intermediate monocytes (CD14+CD16+)** |  |  |  |  | **0.598** | **<0.0001** | **0.545** | **<0.0001** | **0.201** | **<0.0001** | **0.187** | **<0.0001** |
| **% intermediate monocytes (CD14+CD16+)** | **-0.120** | **0.0005** | **-0.157** | **<0.0001** | **0.304** | **<0.0001** | **0.496** | **<0.0001** | **0.192** | **<0.0001** | **0.305** | **<0.0001** |

**Supplementary Tables**

**Table S1 ǀ Association of CD8 population with transplant failure at one year.** CD8 population was analyzed by tercile of CD8 T cell count (A) or percentage of CD8 T cells (B). n patients = 785, missing data = 47, n events = 106.

| CD8 T cells [n] | HR | 95%CI | *p* |
| --- | --- | --- | --- |
| Tercile 1 [39 – 217] | 1 | - | - |
| Tercile 2 [217 – 374] | 1.316 | [0.809; 2.139] | 0.269 |
| Tercile 3 [374 – 1400] | 1.391 | [0.863; 2.244] | 0.176 |

**A**

**B**

| CD8 T cells [%] | HR | 95%CI | *p* |
| --- | --- | --- | --- |
| Tercile 1 [7 – 20 %] | 1 | - | - |
| Tercile 2 [20 – 28%] | 1.004 | [0.623; 1.616] | 0.988 |
| Tercile 3 [28 – 61%] | 1.202 | [0.762; 1.897] | 0.428 |

**Table S2 ǀ Immune population phenotype and description of cluster 2 patients at the day of transplantation among the CMV exposure.**

|  | **CMV-naïve (n=64)** | **CMV-exposed (n=205)** | ***p* value** | | |
| --- | --- | --- | --- | --- | --- |
| **Demographic data** |  |  |  | | |
| Age (years ±SD) | 45 (±15) | 49 (±14) | 0.087 | | |
| Gender (male, %) | 64 | 59 | 0.455 | | |
| **PBMC population** |  |  |  | | |
| CD4 count (n/mm3) | 794 (±334) | 763 (±313) | 0.499 | | |
| CD8 count (n/mm3) | 483 (±147) | 544 (±217) | 0.013 | | |
| CD4 (%) | 47 (±8) | 46 (±8) | 0.539 | | |
| CD8 (%) | 31 (±9) | 33 (±8) | 0.389 | | |
| **T CD4 population % (±SD)** | **n = 38** | **n = 164** |  | | |
| CD4 naïve | 50 (±16) | 43 (±18) | 0.014 |  |
| CD28+ CD4 naïve | 49 (±16) | 42 (±18) | 0.010 | | |
| CD4 CM | 24 (±8) | 26 (±11) | 0.312 | | |
| CD28+ CD4 CM | 24 (±8) | 25 (±11) | 0.597 | | |
| CD4 TEMRA | 6 (±5) | 7 (±6) | 0.242 | | |
| CD28+ CD4 TEMRA | 4 (±5) | 3 (±3) | 0.371 | | |
| CD4 EM | 20 (±12) | 24 (±13) | 0.055 | | |
| CD28+ CD4 EM | 19 (±11) | 20 (±11) | 0.754 | | |
| **T CD8 population % (±SD)** | **n = 38** | **n = 164** |  | | |
| CD8 naïve | 35 (±18) | 27 (±18) | 0.006 | | |
| CD28+ CD8 naïve | 28 (±16) | 20 (±16) | 0.003 | | |
| CD8 CM | 3 (±2) | 3 (±2) | 0.278 | | |
| CD28+ CD8 CM | 2 (±1) | 2 (±2) | 0.659 | | |
| CD8 TEMRA | 42 (±18) | 47 (±16) | 0.059 | | |
| CD28+ CD8 TEMRA | 13 (±8) | 9 (±6) | <0.001 | | |
| CD8 CEM | 20 (±11) | 22 (±12) | 0.170 | | |
| CD28+ CD8 EM | 13 (±9) | 12 (±8) | 0.271 | | |

**Supplementary Figure**

**Figure S1 ǀ Kaplan Meier curves for survival without acute rejection according to number of CD8+ T cells (A) and percentage of CD8+ T cells (B).** (A) Patients were divided into 3 groups based on their CD8 T cell count (tercile): first tercile in red ([39-217] CD8 T cell count), second tercile in green ([217-374] CD8 T cell count) and third tercile in blue ([374-1,4.10^3^] CD8 T cell count). (B) Patients were divided into 3 groups based on their percentage of CD8 T (tercile): first tercile in red ([7%-20%] of CD8 T cell), second tercile in green ([20%-28%] of CD8 T cell) and third tercile in blue ([28%-61%] of CD8 T cell).


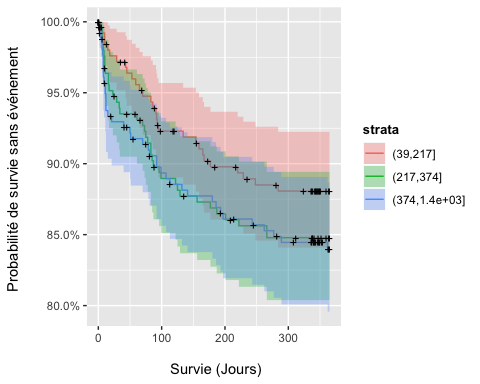
**A**

Survival without rejection

Times (days)

**
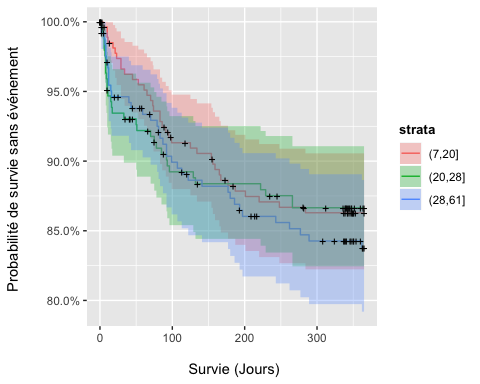
B**

Times (days)

Survival without rejection
